# Significance of baseline neutrophil-to-lymphocyte ratio for progression-free survival of patients with HER2-positive breast cancer treated with trastuzumab emtansine

**DOI:** 10.1038/s41598-018-37633-0

**Published:** 2019-02-12

**Authors:** Michiko Imamura, Takashi Morimoto, Chiyomi Egawa, Reiko Fukui, Ayako Bun, Hiromi Ozawa, Yoshimasa Miyagawa, Yukie Fujimoto, Tomoko Higuchi, Yasuo Miyoshi

**Affiliations:** 1 Department of Surgery, Division of Breast and Endocrine Surgery, Hyogo College of Medicine, Mukogawa-cho 1-1, Nishinomiya, Hyogo 663-8501 Japan; 2 Department of Breast Surgery, Yao Municipal Hospital, Ryuka-cho 1-3-1, Yao City, Osaka 581-0069 Japan; 3 Department of Surgery, Kansai Rosai Hospital, Inabaso 3-1-69, Amagasaki City, Hyogo 660-8511 Japan

## Abstract

The efficacy of trastuzumab emtansine (T-DM1) is prolonged for some patients; however, the predictive factors remain unknown. We focused on a peripheral blood biomarker, the neutrophil-to-lymphocyte ratio (NLR), regarding T-DM1 treatment efficacy. Fifty-three advanced or metastatic breast cancers treated with T-DM1 were retrospectively recruited from three institutes. The NLR in the peripheral blood was measured at baseline and after one cycle. The cutoff value of the NLR was set at median value 2.56. The progression-free survival (PFS) of patients with NLR-low at baseline (n = 26; median, not reached) was significantly better than that of patients with NLR-high (n = 27; median, 4.13 months; hazard ratio [HR], 0.226; 95% confidence interval [CI], 0.112–0.493; p = 0.0001). Longer overall survival was significantly associated with a low NLR (HR, 0.384; 95% CI, 0.170–0.910; p = 0.0296). In the subgroup analysis, patients with NLR-low consistently had longer PFS compared to those with NLR-high irrespective of the number of prior chemotherapy regimens, prior trastuzumab, visceral metastasis, estrogen receptor status, and human epidermal growth factor receptor 2 (HER2) score. Although detailed mechanisms remain unknown, treatment efficacy of T-DM1 may be partly mediated by activation of the immune system. Low baseline NLR appears to be beneficial for treatment with T-DM1 in HER2-positive breast cancers.

## Introduction

Recently, the prognosis of human epidermal growth factor receptor 2 (HER2)-positive locally advanced or metastatic breast cancers (MBCs) has dramatically improved due to the introduction of trastuzumab, pertuzumab, and trastuzumab emtansine (T-DM1)^1^. T-DM1 is an antibody-drug conjugate which combines trastuzumab and the cytotoxic drug DM-1 via a nonreducible thioether linker^2^ which was approved as a second-line or later therapy for HER2-positive MBCs. In the phase III EMILIA clinical trial, progression-free survival (PFS) of patients treated with T-DM1 (median PFS, 9.6 months) was significantly better than that of patients treated with lapatinib plus capecitabine (6.4 months; hazard ratio [HR], 0.65; 95% confidence interval [CI], 0.55–0.77; p < 0.001)^3^. Overall survival (OS) of patients treated with T-DM1 was also significantly superior to that of patients treated with lapatinib plus capecitabine (HR, 0.68; 95% CI, 0.55–0.85; p < 0.001). Similar improvements in PFS and OS were consistently reported by the phase III TH3RESA trial in which HER2-positive MBCs that received two or more HER2-directed regimens were recruited. The PFS of patients receiving T-DM1 was significantly improved compared with the PFS of those assigned a treatment selected by a physician (median, 6.2 months vs. 3.3 months; HR, 0.528; 95% CI, 0.422–0.661; p < 0.0001). In addition, a significantly favorable OS in the T-DM1 group was recognized (HR, 0.552; 95% CI, 0.369–0.826; p = 0.0034)^4^. Furthermore, according to a meta-analysis of five randomized controlled trials of 3720 patients, both PFS (HR, 0.73; 95% CI, 0.61–0.86; p < 0.05) and OS (HR, 0.68; 95% CI, 0.62–0.74, p < 0.05) were significantly improved compared with other anti-HER2 therapies^5^.

The efficacy of T-DM1 has been recognized across all subgroups, including age, estrogen receptor (ER) status, and disease involvement (visceral or non-visceral)^3,4,6^. In addition, exploratory biomarker analysis of the TH3RESA study showed that improved PFS was obtained irrespective of HER3 mRNA levels, phosphatase and tensin homolog (PTEN) H-score, or phosphatidylinositol 4,5-bisphosphate 3-kinase catalytic subunit alpha (PIK3CA) mutation status^7^. Interesting, HR was superior in the higher HER2 mRNA subgroup (above the median) than in the lower HER2 subgroup (at or below the median) (HR, 0.40; 95% CI, 0.28–0.59; p < 0.0001 vs. HR, 0.68; 95% CI, 0.49–0.92; p = 0.0131, respectively). Improved efficacy of T-DM1 in the subgroup of high HER2 expression level was consistently recognized in other reports^8,9^. Accordingly, the benefit of T-DM1 treatment appears to depend on HER2 expression levels in breast cancers. However, biomarkers that predict the treatment efficacy of T-DM1 remain unknown.

In the EMILIA study, the overall response rate in patients with an HER2 mRNA concentration ratio > median (52.8%) was significantly higher than in those ≤median (37.9%; odds ratio, 2.45; 95% CI, 1.58–3.80), and the duration of complete or partial response in the T-DM1 group (median, 12.6 months; 95% CI, 8.4–20.8) was better than that in the lapatinib plus capecitabine group (median, 6.5 months; 95% CI, 5.5–7.2)^3,9^. Interestingly, HER2-positive breast cancer patients with higher intratumor HER2 mRNA levels had lower risk of death when treated with T-DM1 than when with capecitabine plus lapatinib (HR, 0.53; 95% CI, 0.37–0.76). Conversely, there was no OS difference in patients with tumors expression lower HER2 mRNA levels (HR, 0.80; 95% CI, 0.59–1.09); in the case of PFS, the HRs were similar in the case of high or low HER2 mRNA levels (HR, 0.65; 95% CI, 0.50–0.85 and HR, 0.64; 95% CI, 0.50–0.82 for high and low intratumor HER2 mRNA levels, respectively). While specific biological biomarkers, such as intratumor HER2 mRNA levels or HER2 expression, may predict long-term benefit from T-DM1, new, reliable, and easily available predictive factors are required to identify patients more or less likely to benefit from T-DM1. Müller P *et al*. reported that T-DM1 induced antitumor immunity in patients treated with neoadjuvant therapy, with tumor infiltrating lymphocytes (TILs) increasing after the administration of T-DM1^10^. Based on these observations, the benefits of T-DM1 for prognosis may be mediated by an immune reaction against breast cancers, at least in part. As an indicator of cancer immunity, the neutrophil-to-lymphocyte ratio (NLR) has been established in early breast cancers^11–14^. The relationship between low NLR and good prognosis was determined in the ER-negative/HER2-negative (TN) and HER2-positive breast cancers by analysis according to subtypes^12–14^. For recurrent breast cancers, Iwase *et al*. reported that increased NLR was significantly associated with poorer survival^15^. We recently reported the usefulness of the NLR for treatment efficacy in primary advanced and recurrent patients treated with eribulin^16^. In addition, platelet-to-lymphocyte ratio (PLR) was also demonstrated as a significant and independent factor for prognosis of early breast cancer patients^17–19^. Thus, the NLR and PLR may serve as prognostic or predictive indicators of patients treated with T-DM1 although these possibilities have yet to be reported. Therefore, in the present study, we investigated the usefulness of the NLR and PLR for treatment efficacy of T-DM1 in HER2-positive primary advanced and recurrent breast cancers.

## Results

### Clinicopathological characteristics of the NLR-high and -low subsets treated with T-DM1

Among 53 patients treated with T-DM1, 26 and 27 patients were classified into NLR-low (<2.56) and NLR-high (≥2.56), respectively. Frequencies of breast cancers with NLR-low were marginally higher in patients with progesterone receptor-negative, recurrence, and no prior trastuzumab (p = 0.05) (Table 1). There was no significant association between other clinicopathological characteristics, including menopausal status, Eastern Cooperative Oncology Group Performance Status (ECOG PS), disease-free interval, disease control during 1^st^ line treatment, ER status, HER2 IHC score (2+ or 3+), number of metastatic sites, metastatic sites, prior endocrine therapy, brain metastasis, number of prior chemotherapies, prior anthracycline, prior taxene, prior pertuzumab, prior lapatinib, and reason of treatment discontinuation.

**Table 1:** Relationship between neutrophil-to-lymphocyte ratio and clinicopathological characteristics in patients treated with T-DM1.

	n	NLR <2.56^a^	NLR ≥2.56^a^	p-value
**Age (year)**				
Median (Range)	53	60.0 (39–79)	58.4 (43–85)	0.62
**Menopausal status** ^**b**^				
Pre-	4	1 (25.0%)	3 (75.0%)	0.61
Post-	44	22 (50.0%)	22 (50.0%)	
**ECOG PS** ^c^				
0	27	15 (55.6%)	12 (44.4%)	0.41
1 and 2	26	11 (42.3%)	15 (57.7%)	
**Disease-free interval**				
<36 months	10	7 (70.0%)	3 (30.0%)	0.76
≥36 months	11	7 (63.6%)	4 (35.4%)	
**Disease control during 1** ^**st**^ **line treatment** ^**d**^				
< 12 months	33	15 (45.5%)	18 (54.5%)	0.53
≥ 12 months	14	8 (57.1%)	6 (42.9%)	
**Estrogen receptor status**				
Positive	30	13 (43.3%)	17 (56.7%)	0.41
Negative	23	13 (56.5%)	10 (43.5%)	
**Progesterone receptor status**				
Positive	22	7 (31.8%)	15 (68.2%)	0.05
Negative	31	19 (61.3%)	12 (38.7%)	
**HER2 IHC status**				
2+ with FISH+^e^	12	5 (41.7%)	7 (58.3%)	0.74
3+	41	21 (51.2%)	20 (48.8%)	
**Primary advanced or recurrence**				
Primary advanced	32	12 (37.5%)	20 (62.5%)	0.05
Recurrence	21	14 (66.7%)	7 (33.3%)	
**Number of metastatic sites**				
1 and 2	27	15 (56.5%)	12 (43.5%)	0.41
3 and more	26	11 (43.2%)	15 (56.8%)	
**Metastatic sites**				
Non-visceral	16	8 (50.0%)	8 (50.0%)	0.99
Visceral	37	18 (48.6%)	19 (51.4%)	
**Brain metastasis**				
No	41	20 (48.8%)	21 (51.2%)	0.94
Yes	12	6 (50.0%)	6 (50.0%)	
**Prior endocrine therapy**				
Yes	17	7 (41.2%)	10 (58.8%)	0.56
No	36	19 (52.8%)	17 (47.2%)	
**Number of prior chemotherapy**				
0 and 1	26	14 (53.8%)	12 (46.2%)	0.79
2 and 3	18	8 (44.4%)	10 (55.6%)	
4 and more	9	4 (44.4%)	5 (55.6%)	
**Prior anthracycline**				
Yes	8	3 (37.5%)	5 (62.5%)	0.70
No	45	23 (51.1%)	22 (48.9%)	
**Prior taxane**				
Yes	41	20 (48.8%)	21 (51.2%)	0.99
No	12	6 (50.0%)	6 (50.0%)	
**Prior trastuzumab**				
Yes	49	22 (44.9%)	27 (55.1%)	0.05
No	4	4 (100%)	0 (0%)	
**Prior pertuzumab**				
Yes	32	17 (53.1%)	15 (46.9%)	0.58
No	21	9 (42.9%)	12 (57.1%)	
**Prior lapatinib**				
Yes	39	18 (46.2%)	21 (53.8%)	0.54
No	14	8 (57.1%)	6 (42.9%)	
**Reason of treatment discontinuation** ^**f**^				
Disease progression	27	7 (25.9%)	20 (74.1%)	0.44
Adverse events	4	2 (50.0%)	2 (50.0%)	
Others	4	2 (50.0%)	2 (50.0%)	

^a^NLR: neutrophil-to-lymphocyte ratio.

^b^The menopausal status of five cases was unknown.

^c^ECOG PS: Eastern Cooperative Oncology Group Performance Status.

^d^Four cases with no prior chemotherapy and two unknown cases were excluded.

^e^FISH: fluorescence *in situ* hybridization.

^f^18 patients were on treatment.

### Relationship between the NLR or PLR and patient outcomes

**Figure 1:**
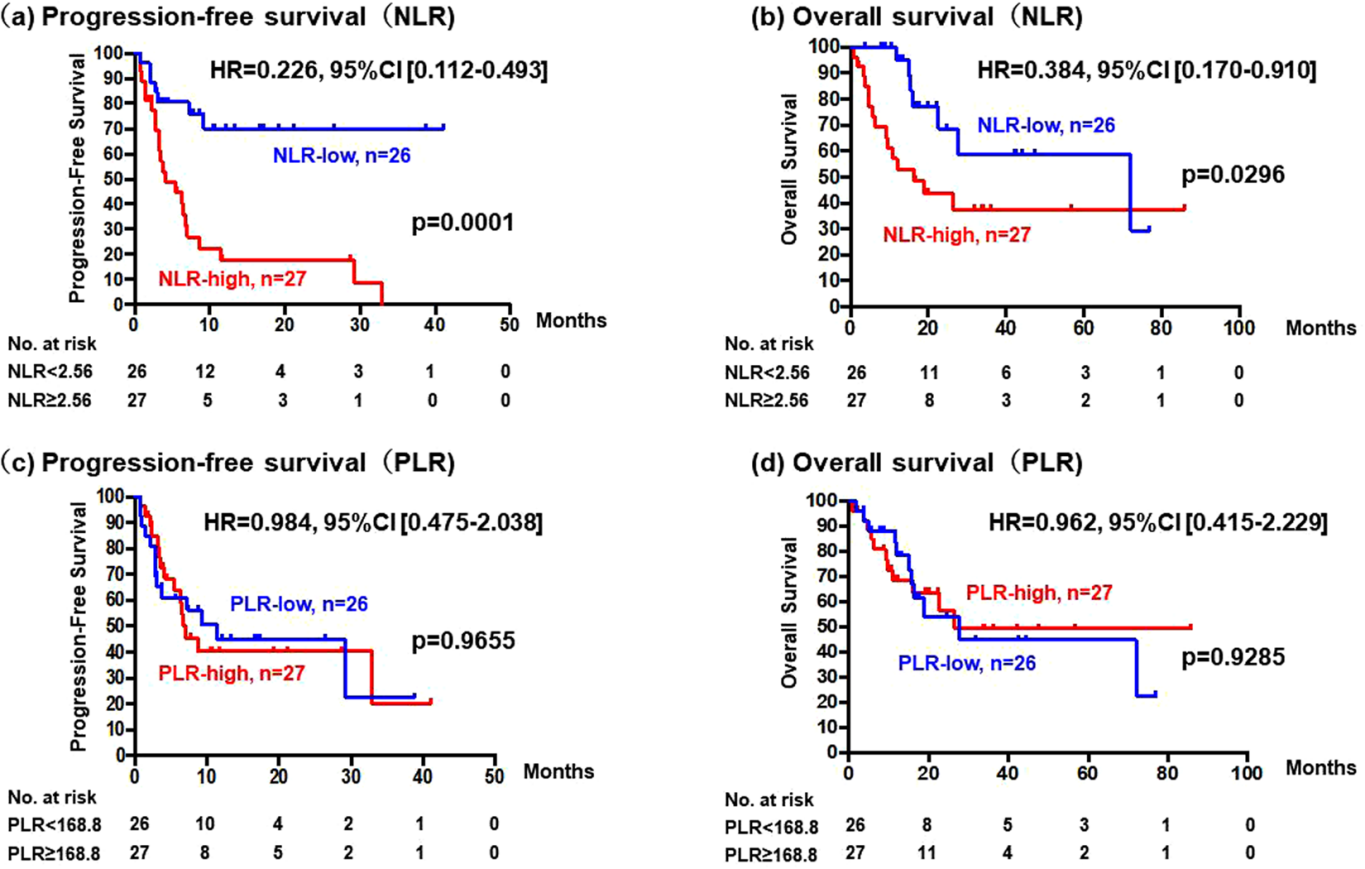
(**a**) Progression-free survival and (**b**) overall survival of patients treated with trastuzumab emtansine (T-DM1) according to baseline levels of the neutrophil-to-lymphocyte ratio (NLR). (**c**) Progression-free survival and (**d**) overall survival of patients according to baseline levels of the platelet-to-lymphocyte ratio (PLR). NLR-low (<2.56, n = 26) and NLR-high (≥2.56, n = 27); PLR-low (<168.8, n = 26) and PLR-high (≥168.8, n = 27).

PFS of patients treated with T-DM1 was significantly better in the NLR-low group (n = 26; median, not reached) than in the NLR-high group (n = 27; median PFS, 4.13 months) (HR, 0.226; 95% CI, 0.112–0.493; p = 0.0001; Fig. 1a). In addition, OS in the NLR-low group (median OS, 72.1 months) was significantly better than in the NLR-high group (median OS, 16.3 months) (HR, 0.384; 95% CI, 0.170–0.910; p = 0.0296; Fig. 1b). In contrast, there was no significant difference in PFS or OS between PLR-high and -low groups (Fig. 1c,d).

In the subgroup analysis, patients in the NLR-low group consistently had an improved PFS compared with those in the NLR-high group irrespective of prior chemotherapy, prior trastuzumab, visceral metastasis, or institutes (Supplementary Fig. 1). Next, we calculated HRs and corresponding 95% CIs according to different NLR cutoff values from 1.5 to 4.0. As shown in Supplementary Fig. 2, the most significant cutoff value was estimated at 2.5, close to the median value 2.56.

### Univariable and multivariable analyses of PFS or OS among patients treated with T-DM1

Univariable analysis of each clinical and biological factor of PFS showed that disease control during 1^st^ line treatment (p = 0.024), number of metastatic sites (p = 0.041), and NLR (p = 0.0001) were significant factors for PFS (Table 2). Multivariable analysis of these factors showed that disease control during 1^st^ line treatment ≥12 months (HR, 0.364, 95% CI, 0.105–0.976; p = 0.044) and NLR-low (HR, 0.271, 95% CI, 0.104–0.626; p = 0.0019) were significantly associated with a favorable PFS. As for OS, ECOG PS (HR, 3.558, 95% CI, 1.435–10.037; p = 0.006), prior taxene (HR, 3.170, 95% CI, 1.026–14.133; p = 0.045), and NLR (HR, 0.347, 95% CI, 0.131–0.837; p = 0.018) were significant and independent factors by multivariable analysis (Table 3).

**Table 2:** Univariable and multivariable analyses of progression-free survival of patients treated with T-DM1.

	Univariable			Multivariable	
	n	HR (95% CI)^a^	p-value	HR (95% CI)^a^	p-value
**Menopausal status**					
Pre-	4	1.000	0.180		
Post-	44	0.387 (0.126–1.682)			
**ECOG PS** ^**b**^					
0	27	1.000	0.135		
1 and 2	26	1.760 (0.839–3.804)			
**Disease-free interval**					
<36 months	10	1.000	0.594		
≥36 months	11	0.722 (0.206–2.426)			
**Disease control during 1** ^**st**^ **line treatment**					
<12 months	33	1.000	0.024	1.000	0.044
≥12 months	14	0.334 (0.098–0.875)		0.364 (0.105–0.976)	
**Estrogen receptor status**					
Positive	30	1.000	0.995		
Negative	23	1.003 (0.472–2.089)			
**HER2 IHC status**					
2+ with FISH+	12	1.000	0.879		
3+	41	0.931 (0.398–2.543)			
**Primary advanced or recurrence**					
Primary advanced	32	1.000	0.424		
Recurrence	21	0.735 (0.346–1.536)			
**Number of metastatic sites**					
1 and 2	27	1.000	0.041	1.000	0.246
3 and more	26	2.162 (1.032–4.673)		1.610 (0.723–3.764)	
**Metastatic sites**					
Non-visceral	16	1.000	0.503		
Visceral	37	1.315 (0.604–3.163)			
**Brain metastasis**					
No	41	1.000	0.589		
Yes	12	1.273 (0.500–2.865)			
**Prior pertuzumab**					
No	21	1.000	0.943		
Yes	32	1.028 (0.481–2.281)			
**Prior lapatinib**					
No	39	1.000	0.545		
Yes	14	1.283 (0.552–2.767)			
**Number of prior chemotherapy**					
0 and 1	26	1.000			
2 and 3	18	0.865 (0.348–2.067)	0.744		
4 and more	9	2.311 (0.899–5.631)	0.080		
**Prior Taxane**					
No	12	1.000	0.462		
Yes	41	1.373 (0.607–3.524)			
**Neutrophil- to-lymphocyte ratio**					
≥2.56	27	1.000	0.0001	1.000	0.0019
<2.56	26	0.226 (0.112–0.493)		0.271 (0.104–0.626)	

^a^Hazard ratio (95% confidence interval).

^b^ECOG PS: Eastern Cooperative Oncology Group Performance Status.

**Table 3:** Univariable and multivariable analyses of overall survival of patients treated with T-DM1.

	Univariable			Multivariable	
	n	HR (95% CI)^a^	p-value	HR (95% CI)^a^	p-value
**Menopausal status**					
Pre-	4	1.000	0.933		
Post-	44	0.939 (0.169–3.706)			
**ECOG PS**					
0	27	1.000	0.006	1.000	0.006
1 and 2	26	3.494 (1.418–9.812)		3.558 (1.435–10.037)	
**Disease-free interval**					
<36 months	10	1.000	0.143		
≥36 months	11	0.379 (0.081–1.372)			
**Disease control during 1** ^**st**^ **line treatment**					
<12 months	33	1.000	0.175		
≥12 months	14	0.452 (0.104–1.378)			
**Estrogen receptor status**					
Positive	30	1.000	0.698		
Negative	23	0.845 (0.346–1.973)			
**HER2 IHC status**					
2+ with FISH+	12	1.000	0.534		
3+	41	0.715 (0.273–2.222)			
**Primary advanced or recurrence**					
Primary advanced	32	1.000	0.461		
Recurrence	21	1.370 (0.593–3.169)			
**Number of metastatic sites**					
1 and 2	27	1.000			
3 and more	26	1.489 (0.641–3.619)	0.356		
**Metastatic sites**					
Non-visceral	16	1.000	0.100		
Visceral	37	2.338 (0.868–8.120)			
**Brain metastasis**					
No	41	1.000	0.715		
Yes	12	1.209 (0.395–3.092)			
**Prior pertuzumab**					
No	21	1.000	0.946		
Yes	32	1.030 (0.435–2.503)			
**Prior lapatinib**					
No	39	1.000	0.481		
Yes	14	1.379 (0.545–3.279)			
**Number of prior chemotherapy**					
0 and 1	26	1.000			
2 and 3	18	1.444 (0.514–4.146)	0.481		
4 and more	9	2.602 (0.878–7.745)	0.083		
**Prior Taxane**					
No	12	1.000	0.032	1.000	0.045
Yes	41	3.324 (1.100–14.42)		3.170 (1.026–14.133)	
**Neutrophil- to-lymphocyte ratio**					
≥2.56	27	1.000	0.0291	1.000	0.018
<2.56	26	0.384 (0.170–0.910)		0.347 (0.131–0.837)	

^a^Hazard ratio (95% confidence interval).

^b^ECOG PS: Eastern Cooperative Oncology Group Performance Status.

### Change in peripheral biomarkers at baseline and after one cycle of T-DM1 treatment

**Figure 2:**
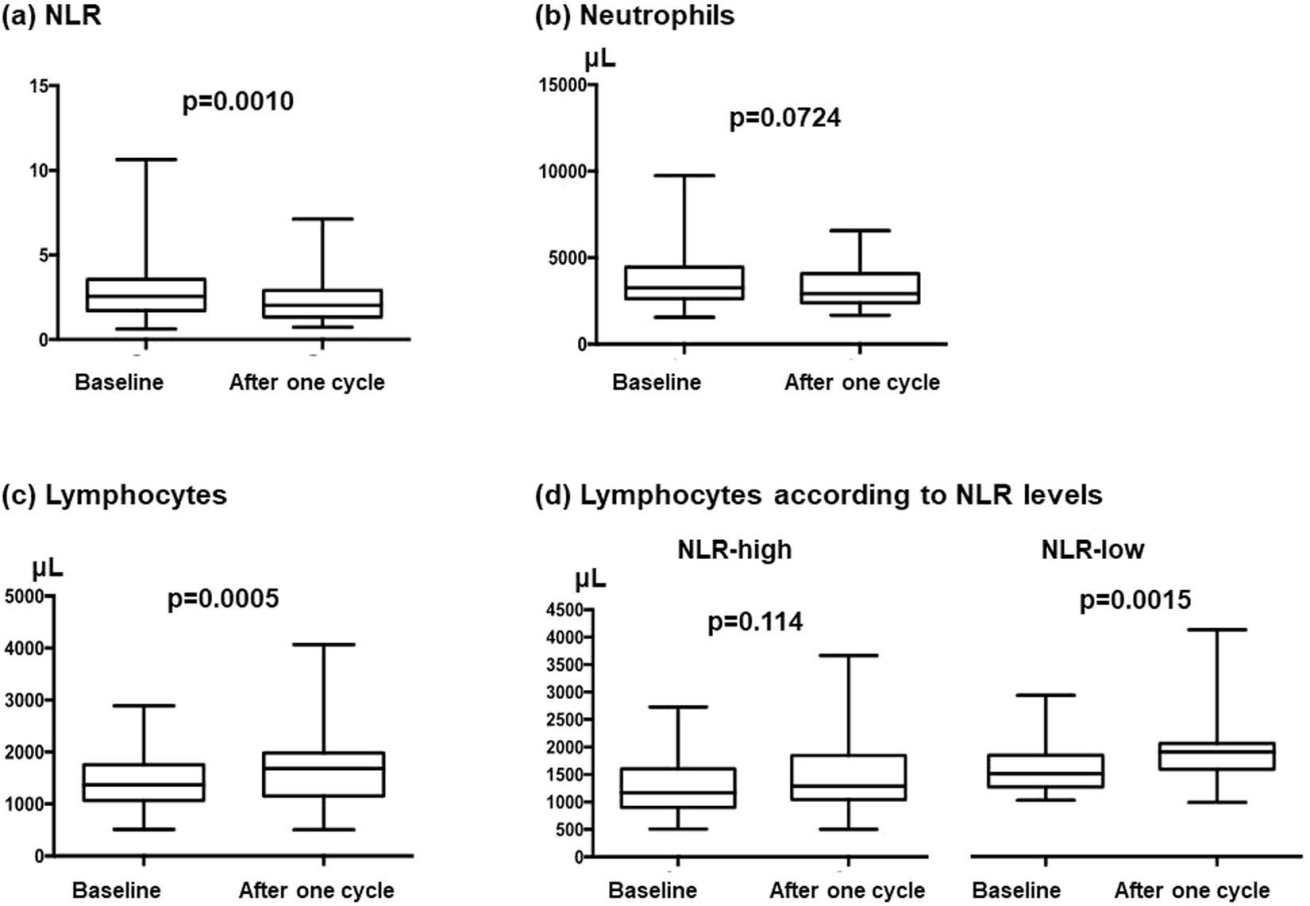
Changes in peripheral blood biomarkers between baseline and after one cycle as indicated by (**a**) neutrophil-to-lymphocyte ratio (NLR), (**b**) neutrophil counts, (**c**) lymphocyte counts, and (**d**) lymphocyte counts according to NLR levels.

The NLR at baseline (median, 2.563; range, 0.634–10.642) was significantly decreased after one treatment cycle (median, 2.037; range, 0.743–7.130; p = 0.0010, Fig. 2a). Although there was no significant difference in neutrophil count at baseline and after one cycle (median, 3231 [range, 1552–9749] vs. 2937 [1672–6559]; p = 0.0724; Fig. 2b), lymphocyte count was significantly increased after one cycle (1367 [508–2888] vs. 1680 [506–4068]; p = 0.0005; Fig. 2c). Interestingly, lymphocyte count was significantly increased in patients in the NLR-low group after one cycle (1488 [1012–2888] vs. 1878 [974.4–4068]; p = 0.0015); in the NLR-high group, lymphocyte count was not significantly different after one cycle of treatment (1168 [509–2727] vs. 1289 [506–3665]; p = 0.114; Fig. 2d).

**Figure 3:**
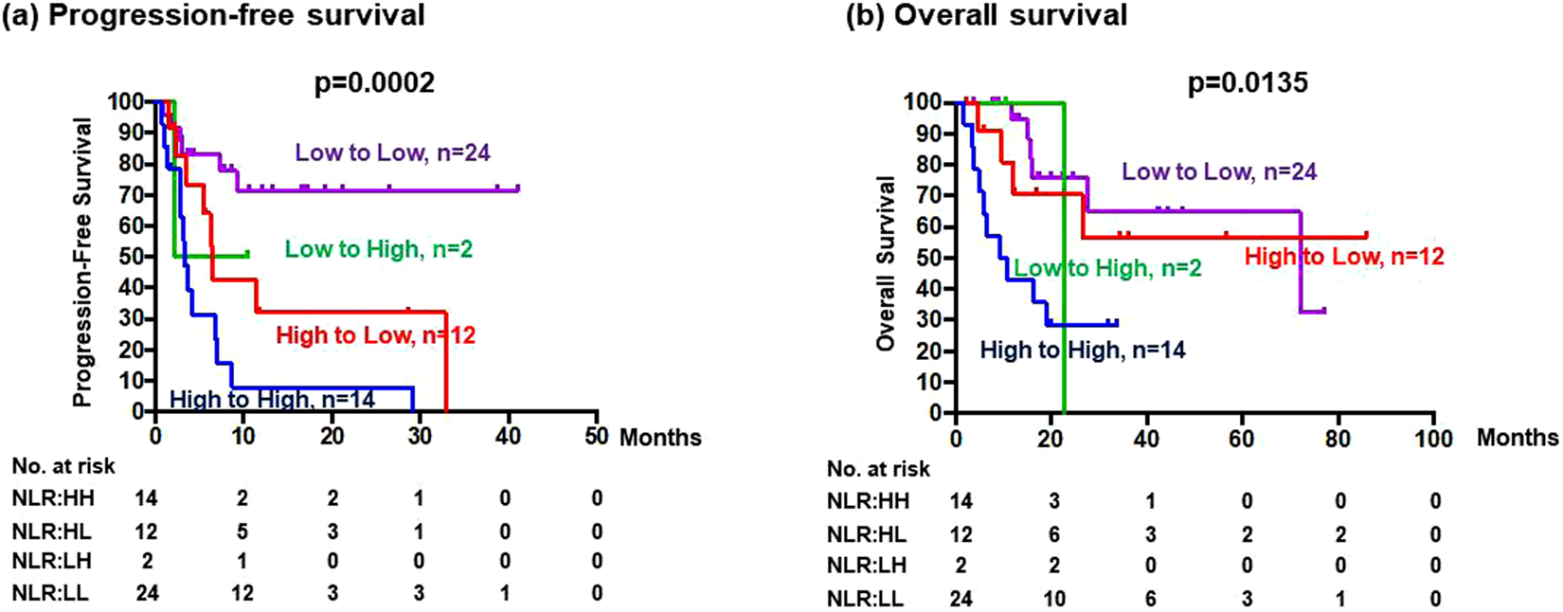
(**a**) Progression-free survival and (**b**) overall survival of patients according to changes of neutrophil-to-lymphocyte ratio (NLR) after one cycle of trastuzumab emtansine (T-DM1) treatment, i.e., low to low (LL, n = 24), low to high (LH, n = 2), high to low (HL, n = 12), and high to high (HH, n = 14). NLR-low < 2.56 and NLR-high ≥ 2.56.

PFS of patients whose NLR was high at baseline but changed to low after one T-DM1 cycle (n = 12; median PFS, 6.47 months) was better than that of patients with a consistently high NLR (n = 14; median PFS, 3.27 months) but worse than that of patients with a consistently low NLR (n = 24; median PFS, not reached; Fig. 3a). On the contrary, OS of patients whose an NLR changed from high at baseline to low after one treatment cycle was similar to that of patients with a consistently low NLR (Fig. 3b).

## Discussion

In the present study, we demonstrated that PFS of patients with a low NLR at baseline was significantly better than that of patients with a high NLR. The association between a low NLR and improved PFS was observed irrespective of ER status (positive or negative), HER2 IHC score (2+ or 3+), or metastatic sites (visceral or non-visceral). Interestingly, the prognosis of patients whose NLR decreased from high to low after one cycle of T-DM1 appeared to be favourable in terms of OS rather than PFS.

The significant association between a high HER2 expression level and improved T-DM1 treatment efficacy has been established^8,9^. Since HER2-bounded T-DM1 is endocytosed and degraded in lysosomes^20^, it is speculated that DM1 concentration is increased in breast cancer cells with high levels of HER2 in the membrane. As mentioned previously, T-DM1 has a more favorable effect on OS than on PFS in patients with high HER2 mRNA levels^9^. In addition, a subgroup of patients with asymptomatic central nervous system metastases at baseline was analyzed in the EMILIA study^21^. Among these patients, significantly improved OS was achieved in the T-DM1 arm compared with the lapatinib-plus-capecitabine arm (HR, 0.382; 95% CI, 0.184–0.795; p = 0.0081), although the PFS was similar (HR, 1.000, 95% CI, 0.542–1.844; p = 0.9998). The mechanisms of T-DM1 that contribute to OS rather than PFS improvement are unlikely to be solely the result of high HER2 expression, and other factors may be involved in this process.

TIL is a simple but useful indicator of immune reaction against cancer cells^22^. In the CLEOPATRA study, which compared pertuzumab or placebo in addition to trastuzumab and docetaxel for HER2-positive MBC, TILs were retrospectively analyzed^23^. In this report, there was no significant association between TIL values (each 10% increment) and PFS (adjusted HR, 0.95; 95% CI, 0.90–1.00; p = 0.063) in all patients. In contrast to PFS, OS was significantly improved (adjusted HR, 0.89; 95% CI, 0.83–0.96; p = 0.0014). Notably, favorable PFS in patients with >20% TILs compared to those with ≤20% TILs was achieved in the pertuzumab group (HR, 0.72; 95% CI, 0.53–0.97; p = 0.029) but not in the placebo group (HR, 0.93; 95% CI, 0.72–1.19, p = 0.55). These data suggest that treatment efficacy of pertuzumab stems, at least in part, from the modulation of immunity in breast cancer. Whether the response induced by T-DM1 correlates with an immune reaction has yet to be studied. In tumor-bearing mice treated with T-DM1, survival was reduced by depleting antibodies which inhibit the function of CD4+ and CD8+ T cells^10^. Based on these results, T-DM1-induced efficacy may be partly mediated through immunity.

Hematologic parameters, including NLR, were not significantly associated with the prognosis of HER2-positive breast cancers^24^. Similarly, Ulas *et al*. reported that in patients with early breast cancer receiving adjuvant trastuzumab, there was no significant association between NLR levels and DFS or OS^25^. These data suggest that NLR is not a sole prognostic factor at least in HER2-positive breast cancers. In addition, predictive usefulness of NLR for chemotherapeutic effects has been demonstrated in breast cancers treated with neoadjuvant chemotherapy for all subtypes^26^ and the TN subtype^27,28^. We previously identified the usefulness of the NLR in HER2-negative primary advanced and recurrent breast cancers treated with eribulin but not with nab-paclitaxel^16^. Similarly, Vernieri *et al*. showed that high NLR was significantly associated with lower PFS in TN breast cancers treated with platinum-containing chemotherapy but this association was not significant in the ER-positive/HER2-negative cancers^29^. These data suggest the predictive value of NLR for a part of treatment efficacy induced by chemotherapy in MBC. Thus, NLR could serve not only as a prognostic but also as a predictive indicator for TN breast cancers, but the significance of NLR still remains unclear in HER2-positive breast cancers.

Lymphocyte counts may reflect an immune reaction or potential immunity against cancer cells. On the contrary, cytokines and chemokines produced by neutrophils play a key role in promoting tumor progression^30,31^. Thus, the NLR, the ratio of these two factors, is considered an indirect indicator for immune reaction, i.e., a low NLR indicates high immunity against cancer cells. This hypothesis may be supported by the report that demonstrated significant associations between the NLR and serum cytokines related to inflammation, including interleukin-6, -8, and -2Rα; hepatocyte growth factor; macrophage-colony stimulating factor; and vascular epidermal growth factor A; in colorectal cancers^32^. In addition, NLR levels were positively associated with the concentration of myeloid-derived suppressor cells in peripheral blood but negatively associated with interferon-γ in breast cancers^33^. Based on these reports, a high NLR may represent an immune suppressive state in the tumor microenvironment. Therefore, we speculate that T-DM1 efficacy may be higher in patients with a low NLR, which reflects lower immune suppression and immune induction related to T-DM1 can be expected. Modulation of the immune reaction by T-DM1 is recognized in the present study by a decrease in the NLR and increased lymphocyte counts after one cycle of treatment (Fig. 2). Although neutrophils appeared to be suppressed due to chemotherapy, a significant increase in lymphocytes after treatment with T-DM1 may indicate direct or indirect immune activation by this drug. Since a significant increase in lymphocytes was recognized in the NLR-low but not in the NLR-high group, we estimated that the induction of lymphocyte-based immunity was possibly inhibited under a high neutrophil concentration. Our data also revealed that the OS of patients whose NLR changed from high to low after one cycle was improved. The observation by Müller P *et al*. that T-DM1 elicits antitumor immunity might be in line with our results^10^.

A limitation of this study is that the NLR cutoff value of 2.56 was a median value. HRs and 95% CIs were calculated using cutoff values ranging from 1.5 to 4.0 (Supplementary Fig. 2) because variable cutoff values from 1.81 to 4.0 are used in early breast cancers^34^. In the present study, the cutoff value at 2.5 seemed to be most significant and, therefore, the median value of 2.56 was considered the best option. The optimal cutoff value needs to be investigated in future studies. Additional limitations of the present study include the small sample size and the short follow-up period. Therefore, the results obtained are not conclusive. However, inconsist with NLR, PLR did not associate with patients’ prognosis, the peripheral immune marker NLR seems to be significant in terms of predicting treatment efficacy obtained from T-DM1. To confirm the results reported here, prospective studies including larger numbers of patients are required.

In conclusion, we have identified that the NLR at baseline might be a significant indicator for efficacy of T-DM1. This significant association was consistently recognized irrespective of ER status or metastatic sites. Since a significant increase in lymphocytes after treatment with T-DM1 in the NLR-low group may indicate immune activation induced by this drug. These findings might contribute to a better understanding of the mechanisms and selecting patients who will benefit from T-DM1.

## Patients and Methods

### Patient eligibility

Primary advanced (n = 32) or recurrent (n = 21) HER2-positive breast cancers treated with T-DM1 between January 2011 and November 2017 were retrospectively and constitutively recruited from three institutes (Hyogo College of Medicine, n = 17; Kansai Rosai Hospital, n = 26; and Yao Municipal Hospital, n = 10). Of all HER2+ breast cancers with primary advanced and recurrence, patients treated with T-DM1 were considered to be eligible for the present study and patients with insufficient clinical data or who discontinued treatment after the first cycle were excluded from the study. The detail profile of patient eligibility selection for this study is described in Supplementary Fig. 3. Histological diagnosis of primary breast cancer was performed in the breast of each patient, and HER2 status was confirmed with an immunohistochemical score (IHC) of 3 or a positive fluorescence *in situ* hybridization test for those with an IHC score of 2, following the American Society of Clinical Oncology/College of American Pathologists HER2 testing in breast cancer guidelines available at that time^35,36^. Metastases were diagnosed using imaging methods such as computed tomography, whole-body bone scintigraphy, or 2-[(18)F]-fluoro-2-deoxy-D-glucose positron emission tomography. Sixteen patients had non-visceral metastases, including bone (n = 7) or locoregional (n = 16), and visceral metastases occurred in 37 patients, including the lung (n = 22), liver (n = 15), brain (n = 12), and pleura (n = 2). All clinical and follow-up data were collected in January 2018.

This study was approved by the ethics committee of the Hyogo College of Medicine (No. 1969) in accordance with the Declaration of Helsinki. As this study collected only retrospective clinical data and offered no risk to the participants, the Institutional Review Board waived the need to obtain written informed consent.

### T-DM1 treatment

T-DM1 was administered intravenously at an initial dose of 3.6 mg/kg every three weeks. Due to adverse events, the dose of T-DM1 was reduced to 3.0 mg/kg in three patients. Concurrent use of denosumab and zoledronic acid took place in 9 and 14 patients, respectively. The median number and range of prior chemotherapies were 2 and 0–9, respectively; 0 (n = 3), 1 (n = 23), 2 (n = 10), 3 (n = 8), and ≥4 (n = 9). Seventeen patients received endocrine therapy for MBC prior to T-DM1. Treatment with T-DM1 continued until disease progression (n = 27), adverse events (n = 4), or other reasons (n = 4), and treatment is ongoing in 18 patients. The median duration of treatment with T-DM1 was 189 days (range, 20–1232 days).

### Measurements of NLR, PLR and patient outcomes

Neutrophil, lymphocyte and platelet counts were determined automatically with Sysmex XN-9000 or XN-1000 hematology analyzers (Sysmex Corporation, Kobe, Japan). We obtained the NLR for each patient by dividing the number of neutrophils (stab and segmented cells) by the number of lymphocytes. A blood test was performed at baseline (the same day just before the start of T-DM1 treatment) and after one cycle of T-DM1 (the same day just before the start of cycle 2) (Supplementary Fig. 3). We used the medians of NLR and PLR as cutoff values as previously reported^25^. The distribution of the NLR at baseline ranged from 0.63 to 10.64, and the median value (2.56) was used as the cutoff value. Similarly, the median of PLR (168.8; range 97.8 to 427.9) was set as the cutoff value. These cutoff values were used to divide patients into different groups: NLR-low (<2.56, n = 26) and NLR-high (≥2.56, n = 27); PLR-low (<168.8, n = 26) and PLR-high (≥168.8, n = 27). PFS was calculated from the start of T-DM1 to the termination of treatment due to disease progression or death from any cause, and OS was calculated from the start of T-DM1 to death from any cause.

### Statistical analyses

The ECOG PS was measured as defined previously^37^. The relationships between the NLR and clinicopathological characteristics were analyzed using Fisher’s exact test or the Wilcoxon rank sum test. Kaplan-Meier plots of PFS or OS in the different groups were calculated using log-rank tests. Univariable analysis of the clinicopathological factors and the NLR was performed using a Cox proportional hazards model to obtain HRs and 95% CIs. Changes in peripheral blood markers at baseline and after one cycle of treatment with T-DM1 were calculated using the Wilcoxon signed rank test. Statistical significance was set at p < 0.05, and statistical calculations were performed using JMP Pro 12 (SAS Institute Inc., Cary, NC, USA).

### Ethical approval

All procedures were performed in accordance with the ethical standards of the institutional research committee and with the 1964 Helsinki Declaration and its later amendments or comparable ethical standards. The study was approved by the ethics committee of the Hyogo College of Medicine (approval number #1969). As this study collected only retrospective clinical data and offered no risk to the participants, the Institutional Review Board waived the need to obtain written informed consent.

## Supplementary information

Supplementary information

## Data Availability

Data are unavailable because the Ethics Committee of the institute did not permit to provide data of individual participants.
