# Supplementary material for: Significance of baseline neutrophil-to-lymphocyte ratio for progression-free survival of patients with HER2-positive breast cancer treated with trastuzumab emtansine
Source: Sci Rep. 2019 Feb 12;9:1811. doi: 10.1038/s41598-018-37633-0 (PMC6372578; doi:10.1038/s41598-018-37633-0)
Supplement: Supplementary file 1 — Supplementary information [file 41598_2018_37633_MOESM1_ESM.pdf]

Supplementary information

Significance of baseline neutrophil-to-lymphocyte ratio for progression-free survival of patients with HER2-positive breast cancer treated with trastuzumab emtansine

Michiko Imamura[1], Takashi Morimoto[2], Chiyomi Egawa[3], Reiko Fukui[1], Ayako Bun[1], Hiromi Ozawa[1], Yoshimasa Miyagawa[1], Yukie Fujimoto[1], Tomoko Higuchi[1], * Yasuo Miyoshi[1*]

1 Department of Surgery, Division of Breast and Endocrine Surgery, Hyogo College of Medicine, Mukogawa-cho 1-1, Nishinomiya, Hyogo 663-8501, Japan

2 Department of Breast Surgery, Yao Municipal Hospital, Ryuka-cho 1-3-1, Yao City, Osaka 581-0069, Japan

3 Department of Surgery, Kansai Rosai Hospital, Inabaso 3-1-69, Amagasaki City, Hyogo 660-8511, Japan

* Correspondence to: Yasuo Miyoshi, Department of Surgery, Division of Breast and Endocrine Surgery, Hyogo College of Medicine, Mukogawa-cho 1-1, Nishinomiya City, Hyogo 663-8501, Japan. Tel: +81-798-45-6374, Fax: +81-798-45-6373, E-mail: ymiyoshi@hyo-med.ac.jp

Supplementary figure 1

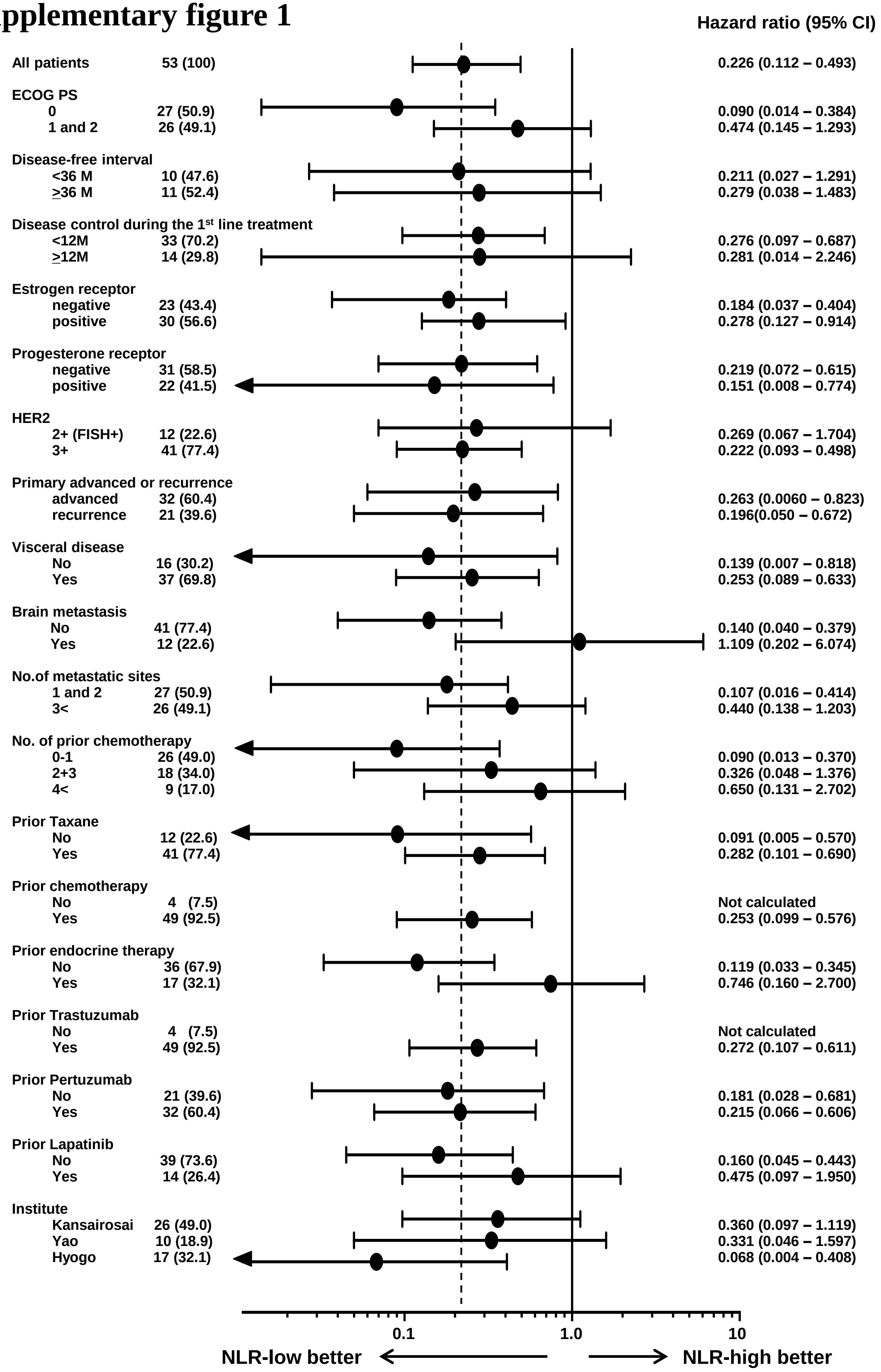

Subgroup analysis of progression-free survival among patients treated with trastuzumab emtansine (T-DM1) according to the neutrophil-to-lymphocyte ratio (NLR). Hazard ratios and 95% confidence intervals in each subgroup are shown. The dashed line shows the hazard ratio (0.226) and 95% confidence interval (0.112-0.493) of all patients.

Supplementary figure 2

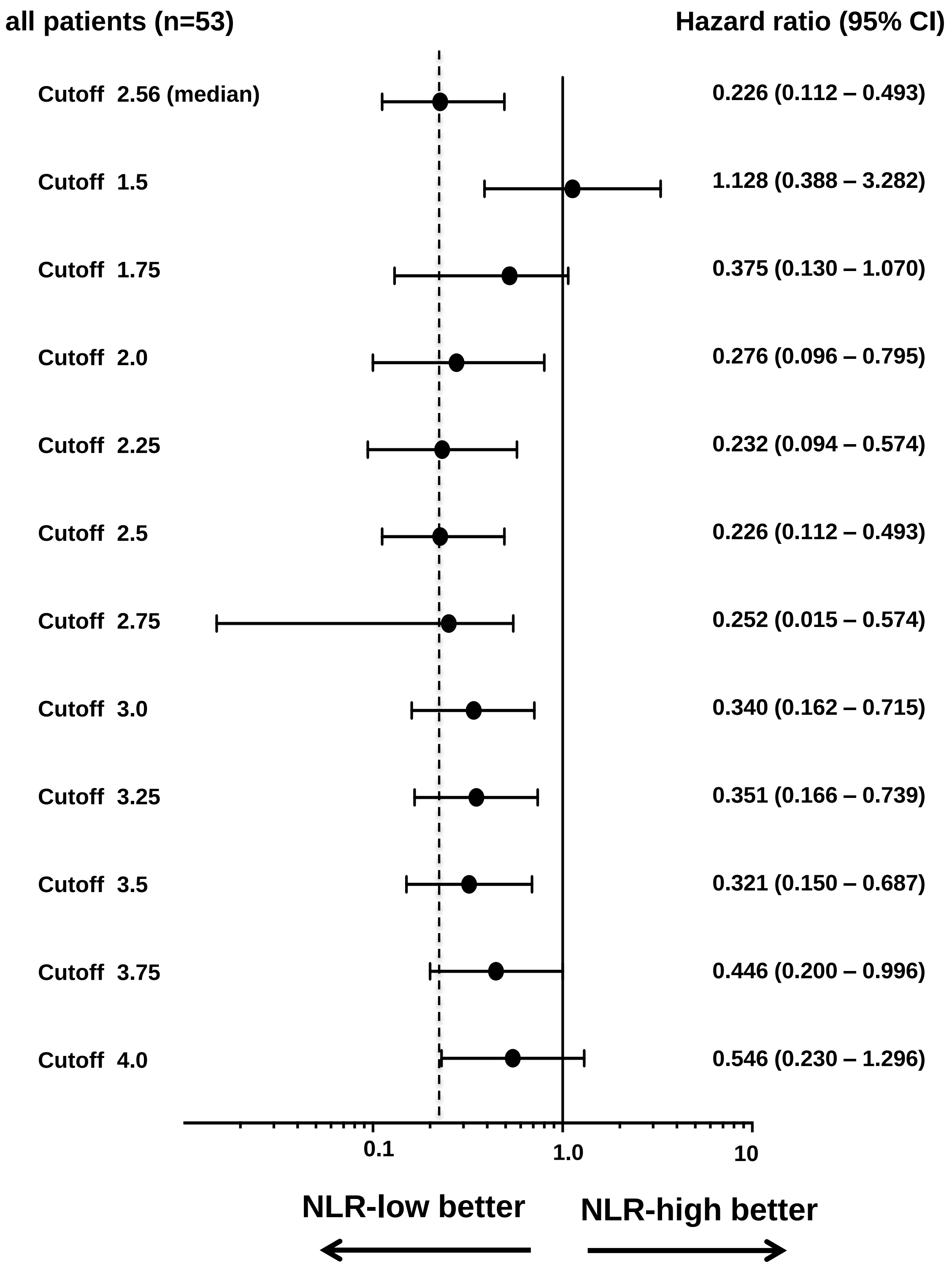

Hazard ratios (HRs) and 95% confidence intervals (CIs) of progression-free survival according to different cutoff values of neutrophil-to-lymphocyte ratio (NLR) from 1.5 to 4.0. The dashed line shows the HR of the median cutoff value of 2.56.

Supplementary figure 3

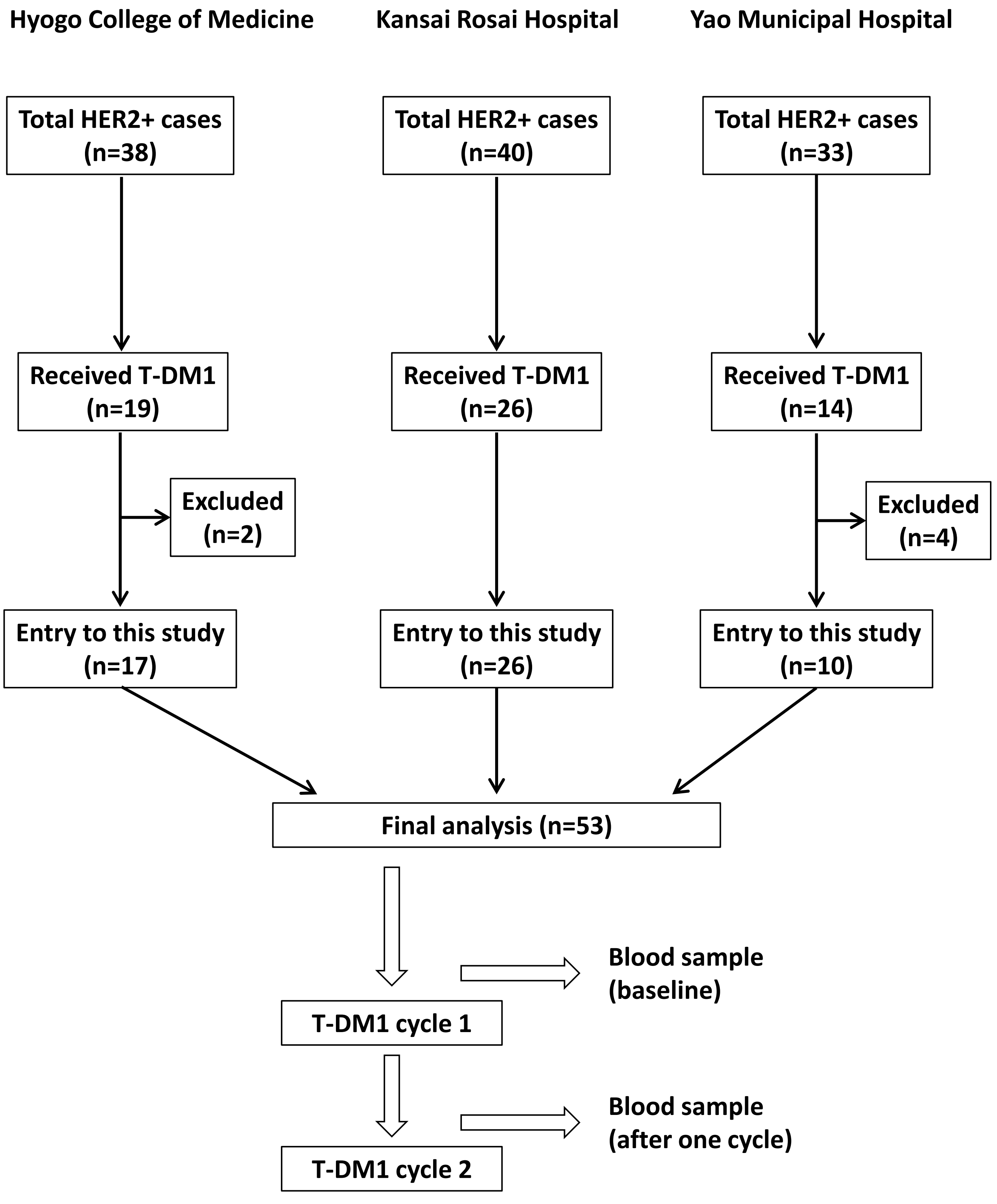

Study diagram of patient eligibility and procedure of blood sampling. Six cases were excluded from the final analyses due to insufficient data or discontinued treatment after one cycle.
